# The Effects of Fisetin on Gene Expression Profile and Cellular Metabolism in IFN-γ-Stimulated Macrophage Inflammation

**DOI:** 10.3390/antiox14020182

**Published:** 2025-02-04

**Authors:** Ziyu He, Xuchi Pan, Kun Xie, Kozue Sakao, Jihua Chen, Masaharu Komatsu, De-Xing Hou

**Affiliations:** 1 The United Graduate School of Agricultural Sciences, Kagoshima University, Kagoshima 890-0065, Japan; k7715393@kadai.jp (Z.H.); sakaok24@agri.kagoshima-u.ac.jp (K.S.); komatsu@fish.kagoshima-u.ac.jp (M.K.); 2 Graduate School of Agriculture, Forestry and Fisheries, Kagoshima University, Kagoshima 890-0065, Japan; 3 Hunan Collaborative Innovation Center for Utilization of Botanical Functional Ingredients, College of Animal Science and Technology, Hunan Agricultural University, Changsha 410128, China; 4 Department of Nutrition Science and Food Hygiene, Xiangya School of Public Health, Central South University, Changsha 410128, China; chenjh@csu.edu.cn

**Keywords:** fisetin, IFN-γ, macrophages, inflammation, RNA-Seq, cellular metabolism, IRF1

## Abstract

Although interferon-gamma (IFN-γ) is known as a critical factor in polarizing macrophages into the pro-inflammatory state for immune response, how dietary flavonoids regulate IFN-γ response for anti-inflammation is incompletely elucidated. This study aims to investigate the effect of fisetin, a typical flavonol, on the inhibition of IFN-γ-induced inflammation by RNA sequencing (RNA-Seq) and cellular metabolism analysis. RAW264 macrophages pretreated with fisetin following IFN-γ stimulation were subjected to RNA-Seq to analyze alterations in gene expression. Cellular signaling and transcription were investigated using enrichment analysis, motif analysis, and transcription factor prediction. Cellular metabolic state was assessed by measuring the oxygen consumption rate (OCR) and lactate level to reflect mitochondrial respiration and glycolysis. Alterations in signaling proteins were confirmed by Western blot. The results revealed that fisetin downregulated the IFN-γ-induced expression of pro-inflammatory genes and M1 marker genes such as *Cxcl9*, *Il6*, *Cd80*, *Cd86*, and *Nos2*. In cellular metabolism, fisetin upregulated the oxidative phosphorylation (OXPHOS) pathway, restored impaired OCR, and reduced lactate production induced by IFN-γ. Motif analysis suggested that fisetin suppressed the activation of IFN-regulatory factor 1 (IRF1). Western blot data further confirmed that fisetin inhibited the phosphorylation of Jak1, Jak2, and STAT1, and decreased the nuclear accumulation of phosphorylated STAT1 and IRF1 induced by IFN-γ. Taken together, our data revealed that fisetin is a potent flavonoid that attenuates IFN-γ-stimulated murine macrophage inflammation and ameliorates disrupted cellular metabolism with a possible Jak1/2-STAT1-IRF1 pathway.

## 1. Introduction

Macrophages are the first line of defense against invading pathogens with high plasticity [1]. Macrophages can be polarized into the classical pro-inflammatory M1 phenotype or the alternative anti-inflammatory M2 phenotype by distinct stimuli. Some inflammatory diseases such as sepsis, inflammatory bowel disease, atherosclerotic disease, and cancer have been reported to relate to the aberrant polarization of macrophages [1,2,3]. Interferons (IFNs) are a group of cytokines classified as type I, type II, or type III interferons. IFN-γ is classified as type II interferon and is primarily secreted by activated T helper 1 cells and natural killer cells [4]. Macrophages are the major targets of IFN-γ, originally identified as the ‘macrophage-activating factor’ that polarizes macrophages to the pro-inflammatory M1 phenotype [5]. IFN-γ is indispensable for host defense against nonviral infections, including bacteria, fungi, and protozoa [6]. IFN-γ also plays a pathogenetic role in hyperinflammatory diseases, including hemophagocytic lymphohistiocytosis, macrophage activation syndrome, and cytokine release syndrome [4,7]. In addition, IFN-γ is the primary effector in rheumatoid arthritis [8], multiple sclerosis [9], systemic lupus erythematosus [10], and other autoimmune diseases [11,12]. Therefore, IFN-γ signaling must be regulated precisely to avoid pathogenetic events.

IFN-γ stimulation can reprogram the profile of cellular gene expression by inducing hundreds of interferon-stimulated genes (ISGs) [6]. These ISGs encode peptides, enzymes, pumps, and channels that restrict pathogens inside macrophages [6]. In IFN-γ signaling, IFN-γ binds to its receptors to induce the activation of tyrosine kinases Jak1, Jak2, and transcription factor STAT1 [13]. The activated STAT1 homodimer is translocated into the nucleus for binding the IFN-γ-activated site (GAS) element in the promoters of ISGs [14]. IFN-regulatory factor 1 (IRF1), a crucial primary ISG induced by STAT1, is a transcription factor that further binds to the interferon-stimulated response element (ISRE) in the promoters of the pro-inflammatory mediators and initiates the expression of inducible nitric oxide synthase (iNOS), cyclooxygenase-2 (COX-2), IFN-β, and interleukin (IL)-12p35 [15,16].

IFN-γ or IFN-γ receptor antibodies and small-molecule inhibitors that target the Jak-STAT pathway are current strategies to suppress IFN-γ signaling [7,17]. However, these strategies have some limitations, such as adverse systemic immune suppression, inconvenient administration, or higher production costs [17,18]. Flavonoids, a family of natural phytochemicals, have been widely studied for their health-promoting and immune-regulating effects, but how flavonoids regulate IFN-γ signaling remains unclear. Fisetin is a common flavonoid found in many natural products like strawberries, chamomile, lime blossom, and green tea [19], and it also can be sustainably supplied through microbial-based biotechnological approaches or chemosynthesis methods [20]. The *in vitro* and *in vivo* anti-inflammatory effects of fisetin have been reported in different models [19,21,22]. Therefore, fisetin was selected as the target molecule to investigate the regulatory effects and molecular mechanisms of IFN-γ signaling through comprehensive approaches in this study.

Human macrophages would be the ideal cell model to investigate the anti-inflammatory effect and molecular mechanism of fisetin. However, there is no definite protocol to differentiate the cells into reproducible macrophage-like cells, although human macrophage-like cells can be derived from primary monocytes or the human monocyte cell line THP-1 by differentiating monocytes [23]. On the other hand, mouse RAW264 cells are differentiated macrophage-like cells and widely used to study immune function and inflammation due to their ability to perform essential macrophage functions and their rapid proliferation as well as ease of handling. Therefore, in this study, we chose mouse RAW264 cells to investigate the anti-inflammatory effect and molecular mechanism of fisetin. Moreover, several lines of previous studies have demonstrated that the pretreatments of fisetin are effective in clarifying the anti-inflammatory activity [24,25,26,27]. Thus, we use pretreatment as a prophylactic design to mimic the daily consumption of plant foods rich in fisetin to prevent inflammation. First of all, RAW264 macrophages pre-treated with fisetin were stimulated by IFN-γ, and total RNA was isolated for RNA sequencing (RNA-Seq) analysis. Differentially expressed genes (DEGs) were then classified by enrichment analysis, and molecular mechanisms were further investigated through motif analysis and transcription factor prediction. Furthermore, mitochondrial respiration capacity was assessed by oxygen consumption rate (OCR) measurement, and glycolytic capacity was evaluated by lactate level. The activation of the Jak1/2-STAT1-IRF1 pathway and the expression of pro-inflammatory mediators were confirmed by Western blot. Our data revealed that fisetin altered gene expression profiling and alleviated cellular metabolism disrupted by IFN-γ in murine macrophages. The inhibition of the Jak1/2-STAT1-IRF1 pathway was suggested to be involved in the molecular mechanisms.

## 2. Materials and Methods

### 2.1. Reagents and Cell Culture

Fisetin (≥99%) was obtained from EXTRASYNTHESE (Genay, Rhône, France) and was dissolved in dimethyl sulfoxide (DMSO). The final concentration of DMSO with or without fisetin was 0.2% in cell culture medium. IFN-γ was purchased from PeproTech, Inc. (Cranbury, NJ, USA), and lipopolysaccharide (LPS, *Escherichia coli* Serotype O55:B5) was from Sigma-Aldrich (St. Louis, MO, USA). The information on antibodies used in this study is listed in Supplementary Table S1. Mouse macrophage-like RAW264 cell line (Cell No. RCB0535) was from RIKEN Bio-Resource Center Cell Bank (Tsukuba, Japan) and was cultured in Dulbecco’s modified eagle medium (DMEM) containing 10% fetal bovine serum (FBS) and 2 mM *L*-glutamine at 37 °C in a 5% CO_2_ atmosphere. In this study, RAW264 cells were used within passages 4 to 15 to ensure consistency and reproducibility of results. Cells exceeding passage 15 were not used; instead, new cultures were initiated from cryopreserved stocks to maintain the passage range between 4 and 15 for all experiments.

### 2.2. RNA Extraction

RAW264 cells (2.74 × 10^5^ cells) at passage 7 were seeded into each well of 6-well plates. After incubation for 21 h, the cells were starved by being cultured in serum-free medium for another 2.5 h to eliminate the influence of FBS. The cells were then treated with or without 5 μM fisetin for 30 min before exposure to 10 ng/mL IFN-γ for 12 h. Total RNA was extracted using the RNeasy Mini Kit (QIAGEN, Hilden, Germany) according to the manufacturer’s manual.

### 2.3. RNA-Seq and Data Analysis

Quality control, cDNA library construction, and sequencing were performed by BGI (Kobe, Japan). Three biological replicates were used for each group. RNA sample quality control was carried out by Fragment Analyzer, and sequencing was executed on the DNBSEQ platform. Clean reads were aligned to the reference genome by HISAT and genes by Bowtie2, respectively. Differential expression was analyzed by the DESeq2 method, and the genes between two groups with adjusted *p*-value (*q*) < 0.05 and fold change (FC) > 1.3 or < 0.77 were considered as differentially expressed genes (DEGs). Data analysis and visualization, including principal component analysis (PCA), Kyoto Encyclopedia of Genes and Genomes (KEGG) enrichment analysis, and Gene Set Enrichment Analysis (GSEA), were carried out by BGI. Motif analysis was performed by Homer software v5. Epigenetic landscape in silico deletion analysis (LISA) for transcription factors inference was performed according to the instructions on the web page [28].

### 2.4. Determination of Oxygen Consumption Rate (OCR)

OCR was determined with an extracellular OCR plate assay kit (Dojindo, Mashiki, Japan) according to the manufacturer’s manual. Briefly, RAW264 cells (2 × 10^4^ cells) were seeded into each well of black clear-bottom 96-well plates. After incubation for 21 h, the cells were starved by being cultured in serum-free medium for another 2.5 h to eliminate the influence of FBS. The cells were then treated with 0, 2.5, or 5 μM fisetin for 30 min before exposure to 10 ng/mL IFN-γ. After a 12 h stimulation, medium containing 10% FBS and an oxygen probe was exchanged into the wells, and the plate was transferred into the microplate reader and incubated at 37 °C for 30 min. Two drops of mineral oil were then added to the wells to prevent air exchange, and fluorescence kinetics (Ex: 500 nm, Em: 650 nm, bottom reading, per 10 min for 200 min) was measured by Infinite 200 PRO MPlex (TECAN Co., Zürich, Switzerland). OCR was calculated using the sheet downloaded from the product page.

### 2.5. Lactate Measurement

Lactate concentration was measured by a Lactate Assay Kit-WST (Dojindo, Japan) according to the manufacturer’s instructions. Briefly, RAW264 cells (2 × 10^4^ cells) were seeded into each well of 96-well plates. After incubation for 21 h, the cells were starved by being cultured in serum-free medium for another 2.5 h to eliminate the influence of FBS. The cells were then treated with 0, 2.5, or 5 μM fisetin for 30 min before exposure to 10 ng/mL IFN-γ for 12 h. The concentration of lactate in the culture medium was determined by measuring absorbance at 450 nm with a microplate reader (Multiscan FC, Thermo Fisher Scientific Inc., Tokyo, Japan).

### 2.6. Western Blot Assay

Western blot assay was performed according to our previous study [29]. Briefly, RAW264 cells were precultured for 21 h and then starved by being cultured in serum-free medium for another 2.5 h to eliminate the influence of FBS. The cells were then treated with 0–20 μM fisetin for 30 min following 40 ng/mL LPS or 10 ng/mL IFN-γ stimulation. After a defined time, the cells were collected and lysed in a lysis buffer (62.5 mM Tris–HCl [pH 6.8], 4% SDS, 100 mM DTT, 10% Glycerol) and boiled for 6–8 min. Equal amounts of protein (10–40 μg) were separated on 8–12% SDS-PAGE and then transferred to a PVDF membrane. After blocking, the membrane was incubated with specific primary antibody at 4 °C overnight and further reacted with corresponding HRP-conjugated secondary antibody at room temperature for 1 h. The bound antibodies were finally detected by ATTO WSE-6170, and the relative amounts of proteins were quantified by ImageJ version 1.54f.

### 2.7. Measurement of Nitric Oxide (NO)

Supernatant nitrite concentration was used to measure NO level by the Griess method [29]. Briefly, RAW264 cells (6 × 10^4^ cells) were seeded into each well of a 48-well plate. After incubation for 21 h, the cells were starved by being cultured in a serum-free medium for another 2.5 h to eliminate the influence of FBS. The cells were then treated with 0–20 μM fisetin for 30 min following 10 ng/mL IFN-γ stimulation. After 12 h, the cell culture medium was collected and reacted with Griess reagent (1% sulfanilamide, 5% phosphoric acid, 0.1% N-[1-naphthyl] ethylenediamide dihydrochloride). The NO concentration was calculated by measuring absorbance at 550 nm (Multiscan FC, Thermo Fisher Scientific Inc., Tokyo, Japan).

### 2.8. Cell Fractionation

Cytoplasmic and nuclear extracts for the Western blot assay were prepared by using the Nuclear/Cytoplasmic Fractionation Kit from Tokyo Chemical Industry Co., Ltd. (Tokyo, Japan). Briefly, RAW264 cells were precultured for 21 h and then starved in a serum-free medium for 2.5 h to eliminate the influence of FBS. The cells were further treated with 0–20 μM fisetin for 30 min following 10 ng/mL IFN-γ stimulation. After 2 h, the cells were collected by 3000× rpm centrifugation for 10 min at 4 °C, and the cellular pellet was suspended in 1× cytoplasmic extraction buffer supplemented with 1 mM DTT, and complete protease inhibitor cocktail, incubated on ice for 10 min, vortexed in the presence of detergents, and centrifuged at 800× *g* for 10 min at 4 °C. The supernatant was collected as a cytoplasmic fraction, and the nuclear pellet was resuspended in the 1× nuclear extraction buffer supplemented with 1 mM DTT and protease inhibitors. The suspension was incubated on ice for 20 min, vortexed every 5 min, and centrifuged for 10 min at 15,000× *g* at 4 °C. The supernatant was collected as a nuclear fraction. The cytoplasmic fractionation and nuclear fractionation were confirmed by Western blot assay.

### 2.9. Statistical Analysis

Results are presented as means ± SD. Significant differences were analyzed by one-way ANOVA followed by Tukey’s multiple range test (SPSS19, IBM Corp., Armonk, NY, USA). *p* < 0.05 was considered statistically significant.

## 3. Results

### 3.1. Fisetin Alters the Gene Expression Profile in IFN-γ-Induced Macrophages

**Figure 1:**
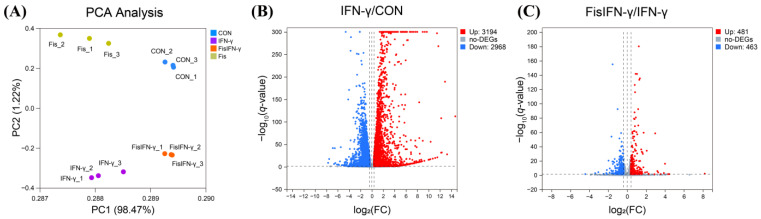
Fisetin altered the gene expression profile in interferon-gamma (IFN)-γ-stimulated macrophages. (**A**) Principal component analysis (PCA) of RNA sequencing (RNA-Seq) data showed that the gene expression profile with or without fisetin pretreatment in IFN-γ-stimulated macrophages formed distinct clusters. (**B**) Volcano plot of the differentially expressed genes (DEGs) in IFN-γ-stimulated cells compared to non-treated cells (IFN-γ/CON). (**C**) Volcano plot of the DEGs in fisetin-pretreated IFN-γ-stimulated cells compared to IFN-γ-stimulated cells (FisIFN-γ/IFN-γ). RAW264 cells were pre-cultured for 21 h and starved in serum-free medium for 2.5 h. The cells were then treated with or without 5 μM fisetin for 30 min and subsequently exposed to 10 ng/mL IFN-γ for 12 h. Total RNA was isolated for RNA-Seq. Fold change (FC) > 1.3 or < 0.77 were identified as DEGs with *q*-value < 0.05. Three biological replicates were used for each group.

To comprehensively understand the effect of fisetin in IFN-γ-polarized macrophages, cellular RNA-Seq was performed. PCA of cellular RNA-Seq data revealed that the gene expression profile with or without fisetin pretreatment in IFN-γ-stimulated macrophages formed distinct clusters (Figure 1A). A total of 14,723 genes were identified from the RNA-Seq and were further classified as DEGs by FC > 1.3 or < 0.77 with *q* < 0.05. IFN-γ-treatment enhanced 3194 DEGs and suppressed 2968 DEGs compared to the control group (Figure 1B and Table 1). Fisetin pretreatment upregulated 481 DEGs and downregulated 463 DEGs in IFN-γ-stimulated macrophages (Figure 1C and Table 2).

### 3.2. Fisetin Attenuates IFN-γ-Induced Pro-Inflammatory Gene Expression

**Figure 2:**
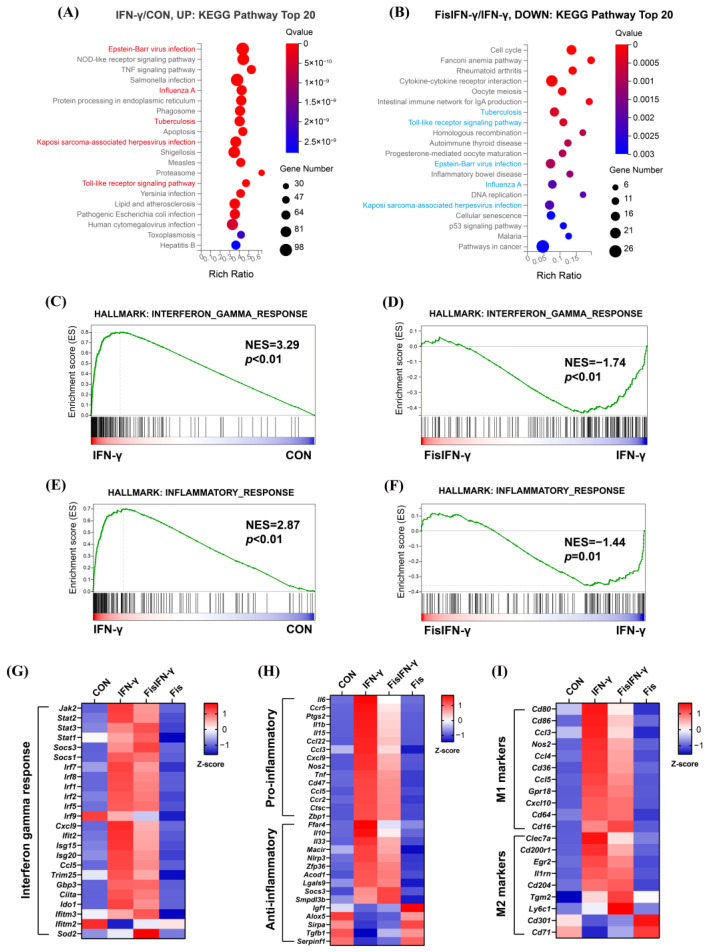
Fisetin inhibited IFN-γ-induced inflammation. (**A**,**B**) Kyoto Encyclopedia of Genes and Genomes (KEGG) pathway enrichment analysis of upregulated DEGs in IFN-γ-stimulated cells compared to non-treated cells (IFN-γ/CON) (**A**) and downregulated DEGs in fisetin-pretreated IFN-γ-stimulated cells compared to IFN-γ-stimulated cells (FisIFN-γ/IFN-γ) (**B**). Red and blue terms represent that the pathway was upregulated by IFN-γ, but downregulated by fisetin pretreatment. (**C**,**D**) Gene Set Enrichment Analysis (GSEA) plots of the total altered genes in interferon gamma response gene set in IFN-γ-stimulated cells compared to control without treatment (**C**) and in fisetin-pretreated IFN-γ-stimulated cells compared to IFN-γ-stimulated cells (**D**). (**E**,**F**) GSEA plots of the total altered genes in inflammatory response gene set in IFN-γ-stimulated cells compared to control without treatment (**E**) and in fisetin-pretreated IFN-γ-stimulated cells compared to IFN-γ-stimulated cells (**F**). The gene sets were from mouse-ortholog hallmark gene sets of the molecular signatures database. (**G**–**I**) Expression heatmaps of representative genes from interferon gamma response gene set (**G**), representative pro-inflammatory and anti-inflammatory genes (**H**), as well as representative genes from macrophages M1 and M2 markers (**I**). Color gradient reflects row Z-score.

To classify the functions of identified DEGs, enrichment analyses were performed. KEGG pathway enrichment analysis revealed that IFN-γ-stimulated DEGs were enriched in pathways related to viral infection, immune response, and diseases, including Epstein-Barr virus infection, toll-like receptor signaling pathway, and tuberculosis (Figure 2A). Fisetin downregulated these terms (Figure 2B). GSEA data revealed that the IFN-γ-enriched interferon-gamma response gene set and the inflammation response gene set were significantly suppressed by fisetin (Figure 2C–F). The expression profile of representative genes in the interferon-gamma response gene set is shown in Figure 2G. Expression heatmaps of pro- and anti-inflammatory genes, as well as M1 and M2 macrophage marker genes, are shown in Figure 2H,I. The expression of pro-inflammatory genes, including *Il6*, *Ptgs2*, *Il1b*, and *Cxcl9*, was significantly inhibited by fisetin, while only a few anti-inflammatory genes, such as *Igf1* and *Alox5*, were enhanced by fisetin. Similarly, fisetin significantly reduced the expression of M1 marker genes, including *Cd80*, *Cd86*, *Nos2*, and *Cd36*, while only inducing the expression of several M2 marker genes such as *Ly6c1* and *Cd71*. Other representative M2 marker genes like *Arg1*, *Mrc1*, *Fizz1*, and *Ym1* were undetected. Taken together, these data showed that fisetin significantly inhibited the IFN-γ-induced inflammatory response at the transcriptional level. Corresponding to this, fisetin inhibited the shift of macrophages to the M1 phenotype under IFN-γ-stimulation, rather than inducing macrophages to the anti-inflammatory M2 phenotype.

### 3.3. Fisetin Regulates Cellular Metabolism in IFN-γ-Stimulated Macrophages

**Figure 3:**
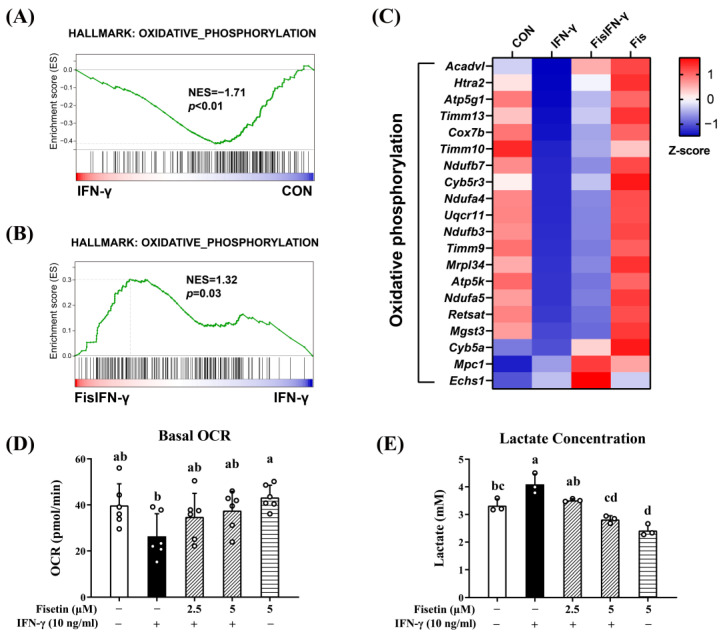
Fisetin modulated metabolism in IFN-γ-stimulated macrophages. (**A**,**B**) GSEA plot of oxidative phosphorylation (OXPHOS) gene set in IFN-γ-stimulated cells compared to non-treated cells (**A**) and in fisetin-pretreated IFN-γ-stimulated cells compared to IFN-γ-stimulated cells (**B**). The gene set was from mouse-ortholog hallmark gene sets of the molecular signatures database. (**C**) Expression heatmap of representative genes from OXPHOS gene set. Color gradient reflects row Z-score. (**D**) Fisetin rescued impaired basal oxygen consumption rate (OCR) in IFN-γ-induced macrophages (*n* = 6). (**E**) Fisetin decreased IFN-γ-enhanced lactate production (*n* = 3). For (**D**,**E**), RAW264 cells were pre-cultured for 21 h and starved in serum-free medium for 2.5 h. The cells were then treated with 0–5 μM fisetin for 30 min and subsequently exposed to 10 ng/mL IFN-γ for 12 h. The cells were used for OCR measurement, and the culture medium was collected for lactate determination. Each value represents the mean ± SD; different letters between groups indicate significant differences (*p* < 0.05). For OCR and lactate determination, at least two separate experiments were performed.

Metabolism in M1 macrophages is characterized by impaired oxidative phosphorylation (OXPHOS) and enhanced aerobic glycolysis [30]. The metabolic state of macrophages treated with fisetin and/or IFN-γ was further investigated. GSEA data showed that the *OXPHOS* gene set was downregulated by IFN-γ and upregulated by fisetin (Figure 3A,B). Representative genes upregulated by fisetin are shown in Figure 3C. Mitochondrial OCR, an index reflecting cellular aerobic respiration, was inhibited by IFN-γ but alleviated by fisetin (Figure 3D). Lactate, the final product of glycolysis, was used to assess glycolysis capacity [31]. The IFN-γ-increased lactate production was suppressed by fisetin (Figure 3E). These data suggest that fisetin rescued impaired OXPHOS and ameliorated glycolysis to modulate metabolism in IFN-γ-stimulated macrophages.

### 3.4. Fisetin-Downregulated Genes Are Enriched to IRF1

**Figure 4:**
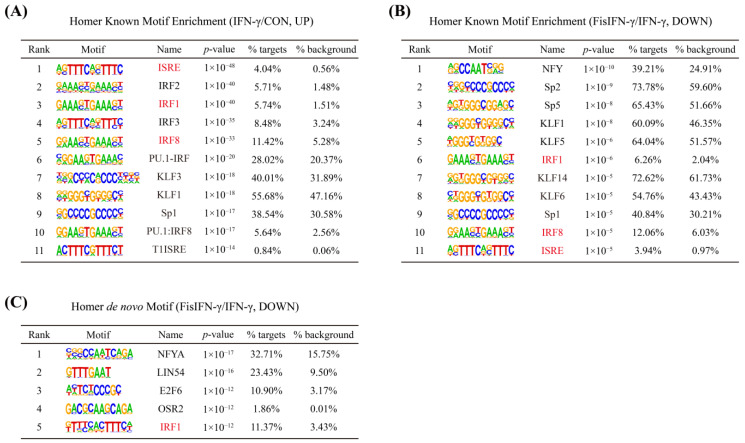
Motif ISRE and IRF1 presented in the promoters of fisetin-downregulated DEGs. (**A**) Homer known motif enrichment results of IFN-γ-upregulated DEGs compared to non-treated cells (IFN-γ/CON, UP). Top 11 motifs were shown. (**B**) Homer known motif enrichment results of fisetin-downregulated DEGs compared to IFN-γ-stimulated cells (FisIFN-γ/IFN-γ, DOWN). Top 11 motifs were shown. (**C**) Homer *de novo* motif enrichment results of fisetin-downregulated DEGs compared to IFN-γ-stimulated cells (FisIFN-γ/IFN-γ, DOWN). The generated 5 motifs were shown. The motifs colored in red are enriched in both IFN-γ-upregulated and fisetin-downregulated DEGs.

To clarify the anti-inflammatory mechanisms of fisetin, the involved promoter motifs and transcription factors were predicted using DEGs. Homer’s known motif enrichment analysis based on 440 reported motifs revealed that 76 motifs were significantly (*p* < 0.01) enriched by IFN-γ-upregulated DEGs, in which ISRE and IRF1 ranked at first and third, respectively (Figure 4A). These two motifs were also significantly enriched by fisetin-downregulated DEGs in IFN-γ-stimulated macrophages, which ranked within the top 11 of the 38 significant motifs (*p* < 0.01) (Figure 4B). In addition, Homer *de novo* motif analysis, using fisetin-downregulated DEGs in IFN-γ-stimulated macrophages, generated 5 motifs, including IRF1 (Figure 4C).

**Figure 5:**
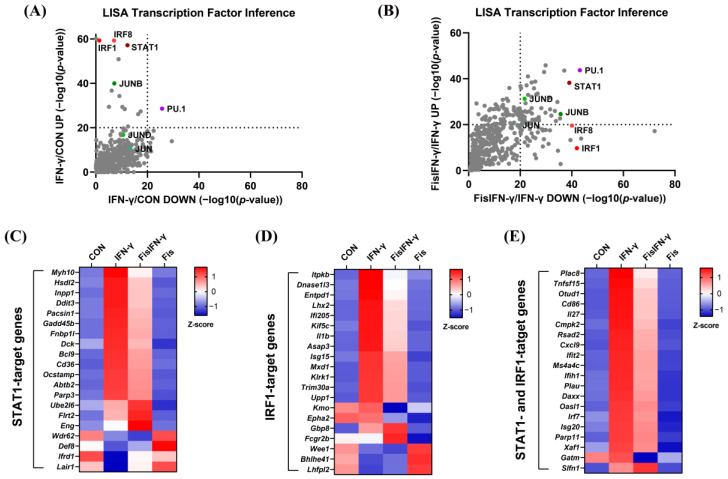
Fisetin-downregulated genes were associated with IFN-regulatory factor1 (IRF1). (**A**,**B**) Transcription factor inference by epigenetic landscape in silico deletion analysis (LISA) on the top 500 DEGs that were upregulated (Vertical axis) or downregulated (Horizontal axis) by IFN-γ, compared to non-treated cells (**A**), and by fisetin pretreatment, compared to IFN-γ-stimulated cells (**B**). (**C**–**E**) Expression heatmaps of the representative genes targeted by STAT1 (**C**), IRF1 (**D**), or both STAT1 and IRF1 (**E**). Color gradient reflects row Z-score.

Next, LISA analysis was performed to detect the transcription factors based on the DEGs. The data revealed that IRF1, IRF8, STAT1, and JUNB were the top transcription factors for the top 500 DEGs, which were upregulated (Vertical axis) or downregulated (Horizontal axis) by IFN-γ, compared to non-treated cells (Figure 5A). In fisetin-pretreated IFN-γ-stimulated macrophages, IRF1 was predominantly associated with the decreased DEGs, and STAT1 was associated with both the decreased DEGs and the increased DEGs (Figure 5B). The expression profiles of representative genes targeted by STAT1, IRF1, or both STAT1 and IRF1 [32,33] are shown in Figure 5C–E. These results suggested that IRF1 might be a master target for fisetin to prevent IFN-γ-induced inflammation.

### 3.5. Fisetin Inhibits IFN-γ-Induced Pro-Inflammatory Mediators Also at Protein Levels

**Figure 6:**
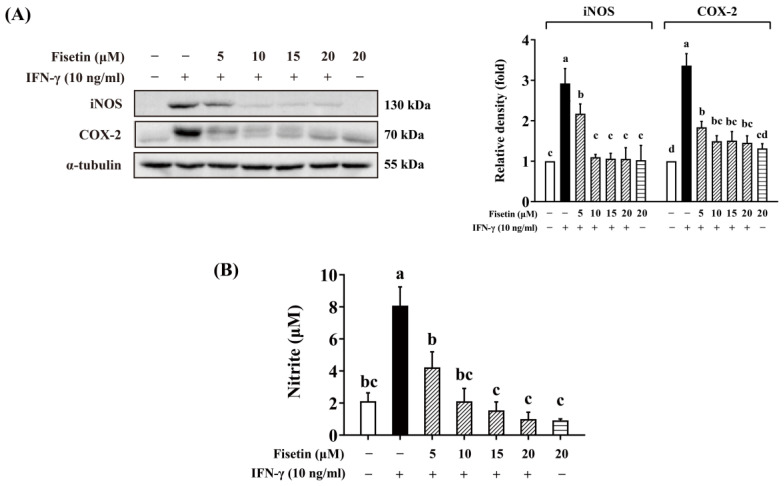
Fisetin suppressed IFN-γ-induced pro-inflammatory mediators at protein levels. (**A**) Fisetin repressed IFN-γ-induced inducible nitric oxide synthase (iNOS) and cyclooxygenase-2 (COX-2) expression in a dose-dependent manner. (**B**) Fisetin dose-dependently inhibited IFN-γ-induced nitric oxide (NO) production (*n* = 3). RAW264 cells were pre-cultured for 21 h and starved in serum-free medium for 2.5 h. The cells were treated with 0–20 μM fisetin for 30 min and then exposed to 10 ng/mL IFN-γ for 12 h. Whole-cell lysates were harvested for Western blot assay, and the culture medium was collected for NO determination. The relative density was calculated as the intensity of the treatment relative to that of the control normalized to α-tubulin by densitometry. Each value represents the mean ± SD; different letters between groups indicate significant differences (*p* < 0.05). The blots presented are representatives from at least three independent experiments, using cells derived from at least two separate preparations. For NO determination, at least two separate experiments were performed.

iNOS and COX-2 are typical pro-inflammatory mediators, and both *Nos2* and *Ptgs2* were identified as fisetin-downregulated DEGs in RNA-Seq. To confirm these results at the protein level, these proteins were further detected by Western blot. As shown in Figure 6A, fisetin significantly inhibited the expression of both iNOS and COX-2 and reduced the level of NO in a dose-dependent manner (Figure 6B). These results confirmed the anti-inflammatory effect of fisetin in IFN-γ-stimulated macrophages at protein levels, which are consistent with the RNA-Seq results.

### 3.6. Fisetin Inhibits Jak1/2-STAT1-IRF1 Pathway

**Figure 7:**
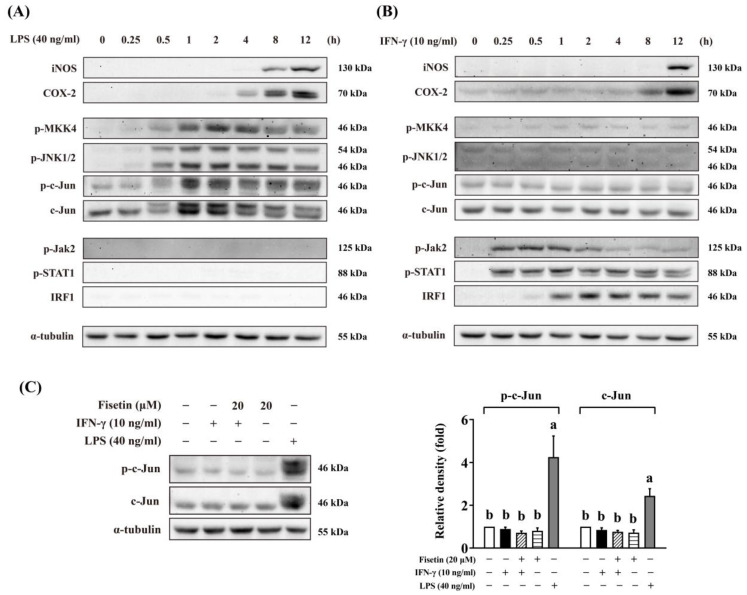
IFN-γ did not activate MAPK pathway. (**A**,**B**) Time-course experiment showed that LPS activated MKK4-JNK-AP-1 cascade with no effect on Jak2-STAT1-IRF1 pathway (**A**), while IFN-γ activated Jak2-STAT1-IRF1 pathway with no effect on MKK4-JNK-AP-1 cascade (**B**). (**C**) IFN-γ did not activate AP-1 (p-c-Jun). RAW264 cells were pre-cultured as described above. The cells were then treated with or without 20 μM fisetin for 30 min and subsequently exposed to 40 ng/mL LPS or 10 ng/mL IFN-γ. Whole-cell lysates were harvested after a defined stimulation time (**A**,**B**) or 30 min (for c-Jun and p-c-Jun in (**C**)), and analyzed by Western blot assay. The relative density was calculated as the intensity of the treatment relative to that of the control normalized to α-tubulin or respective total proteins by densitometry. The bands of total proteins for (**A**,**B**) and semi-quantitative graphs are given in Supplementary Figure S1. Each value represents the mean ± SD; different letters between groups indicate significant differences (*p* < 0.05). The blots presented are representatives from at least three independent experiments, using cells derived from at least two separate preparations.

Fisetin has been found to selectively target MKK4 to inhibit the JNK-AP-1 cascade in LPS-stimulated RAW264 macrophages in our recent study [34]. Since JUNB was enriched in the IFN-γ-induced DEGs in transcription factor inference (Figure 5A), we investigated whether IFN-γ activates the MAPK pathway. A time-course study revealed that LPS activated the MKK4-JNK-AP-1 cascade but did not provoke the Jak2-STAT1 pathway or induce the expression of IRF1 (Figure 7A and Figure S1). In contrast, IFN-γ clearly phosphorylated Jak2 and STAT1 from 15 min and further induced the expression of IRF1 from 1 h. On the other hand, IFN-γ did not activate the MKK4-JNK-AP-1 cascade (Figure 7A–C and Figure S1). These data showed that LPS and IFN-γ elicited distinct pathways for polarizing RAW264 macrophages, and IFN-γ might not trigger the MKK4-JNK-AP-1 cascade.

**Figure 8:**
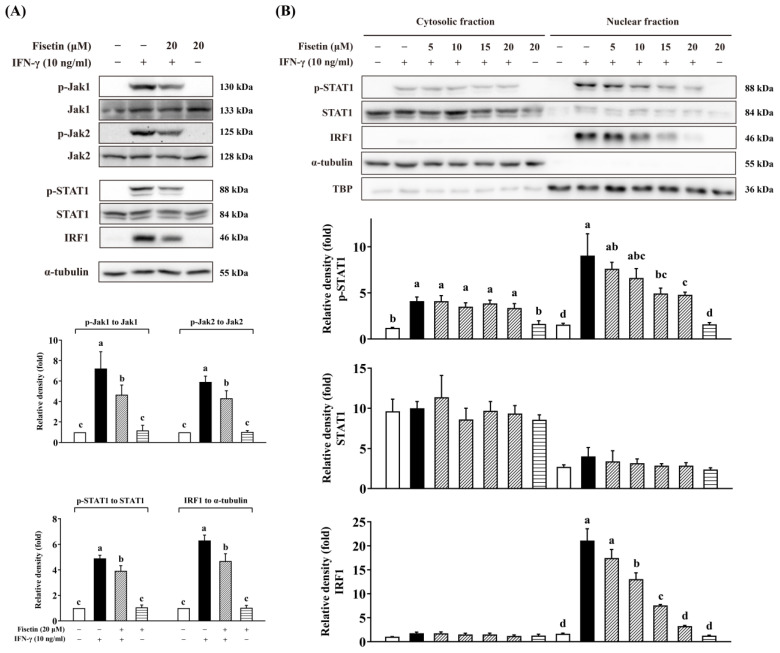
Fisetin inhibited Jak1/2-STAT1-IRF1 pathway. (**A**) Fisetin inhibited phosphorylation of Jak1, Jak2, and STAT1, and the expression of IRF1 induced by IFN-γ. (**B**) Fisetin reduced nuclear accumulation of p-STAT1 and IRF1 in a dose-dependent manner. RAW264 cells were pre-cultured as described above. The cells were then treated with 0–20 μM fisetin for 30 min and subsequently exposed to 10 ng/mL IFN-γ. Whole-cell lysates were harvested after 30 min (for p-Jak1, Jak1, p-Jak2, Jak2, p-STAT1, and STAT1 in (**A**)) or 2 h (for IRF1 in (**A**,**B**)), and then analyzed by Western blot assay. Nuclear and cytoplasmic fractionation was performed as described in Materials and Methods. The relative density was calculated as the intensity of the treatment relative to that of the control normalized to respective total proteins, α-tubulin (for cytoplasmic fraction), or TBP (for nuclear fraction) by densitometry. Each value represents the mean ± SD; different letters between groups indicate significant differences (*p* < 0.05). The blots presented are representatives from at least three independent experiments, using cells derived from at least two separate preparations.

The effect of fisetin on the cellular signaling of the IFN-γ-activated Jak1/2-STAT1-IRF1 pathway was further investigated by Western blot. Fisetin inhibited the phosphorylation of Jak1, Jak2, and STAT1 as well as the expression of IRF1 (Figure 8A).

It has been reported that STAT1 was phosphorylated at Tyr701 by Jak1/2 and then was translocated into the nucleus to induce the expression of ISGs, including IRF1, which subsequently binds to DNA to initiate the expression of pro-inflammatory mediators [4]. To confirm the effect of fisetin on IFN-γ-induced nuclear accumulation of STAT1 and IRF1, we performed nuclear/cytoplasmic fractionation to detect their localization after treatment with fisetin. As shown in Figure 8B, fisetin dose-dependently reduced the nuclear accumulation of p-STAT1 and IRF1. These data suggest that fisetin inhibited the Jak1/2-STAT1-IRF1 pathway to alleviate IFN-γ-induced macrophage inflammation.

## 4. Discussion

Fisetin has been reported to inhibit IFN-γ-stimulated inflammation [35] and ameliorate IFN-γ-related diseases, including systemic lupus erythematosus [36], sclerosis [37], and rheumatoid arthritis [38], in *in vivo* models. However, a thorough understanding of how fisetin regulates the IFN-γ response is still elusive. To clarify the inhibitory effect and molecular mechanism of fisetin in IFN-γ-polarized macrophages, cellular RNA-Seq was performed in this study. Enrichment analysis revealed that fisetin inhibited IFN-γ-triggered immune responses and inflammatory signaling pathways. It is proposed that fisetin primarily suppresses cellular inflammation by downregulating the expression of pro-inflammatory and M1 marker genes, rather than enhancing the expression of anti-inflammatory and M2 marker genes (Figure 2). Notably, the expression of *Acod1*, the gene that encodes the enzyme ACOD1 to produce the critical anti-inflammatory metabolite itaconate [39,40], was significantly inhibited by fisetin (*q* < 0.05, FC > 0.77). Consequently, it is considered unlikely that the suppressed inflammation and modulated cell metabolism would be sustained by the translated products of the genes regulated by fisetin-induced gene expression changes.

To elucidate the anti-inflammatory mechanism of fisetin at the transcriptional level, motif analysis, and transcription factor prediction were performed based on fisetin and/or IFN-γ-caused DEGs. STAT1, IRF1, IRF8, and PU.1 have been reported as the primary transcription factors to regulate the IFN-γ response. Among these, the constitutive binding of IRF8 and PU.1 to DNA was hardly affected by IFN-γ, whereas IRF1 and STAT1 were strongly recruited to cis-regulatory elements in response to IFN-γ stimulation [41]. Our data showed that ISRE and IRF1 motifs, as well as STAT1 and IRF1 transcription factors, were noticeably enriched in IFN-γ-induced DEGs. The IRF1 motif was enriched in fisetin-downregulated genes through both known motif and *de novo* motif analyses. Similarly, IRF1 was enriched in fisetin-downregulated DEGs, and STAT1 was enriched in both fisetin-upregulated and -downregulated DEGs. These findings suggested that the anti-inflammatory effect of fisetin in IFN-γ-treated macrophages may primarily depend on its regulation of IRF1. IRF1 is a GAS-containing gene, and its transcription is STAT1-dependent [6,42,43]. Our data revealed that fisetin inhibited the expression and nuclear accumulation of IRF1 by inhibiting the phosphorylation of Jak1, Jak2, and STAT1, and further the nuclear accumulation of p-STAT1 and IRF1. These results agreed with the reports of some flavonoids that have been reported to regulate IFN-γ signaling through the inhibition of the Jak1/2-STAT1-IRF1 pathway [44]. For example, quercetin bound and inhibited Jak2 [45]. Moreover, quercetin prevented the phosphorylation of STAT1 to decrease IFN-γ-induced iNOS expression and NO production in RAW264.7 cells [46]. Myricetin was found to directly bind to Jak1 [47] and has been reported to prevent the phosphorylation of STAT1 and inhibit both the expression and nuclear translocation of IRF1 to suppress IFN-γ-induced IDO1 expression in human lung cancer cells [48]. Our results are also consistent with previous research showing that fisetin inhibited the phosphorylation of Jak1, Jak2, and STAT1 in LPS/IFN-γ-induced microglial cells [35]. Taken together, our data suggested that fisetin might ameliorate IFN-γ-stimulated macrophage inflammation by inhibiting Jak1/2-STAT1-IRF1 signaling.

Impaired mitochondrial OXPHOS and enhanced aerobic glycolysis occur in M1 macrophages [49,50]. Metabolic regulation of macrophages is considered a strategy to ameliorate inflammation [31,51]. Several studies have reported that flavonoids have the potential to regulate metabolism in macrophages by modulating metabolite-related gene expression [52], regulating metabolism enzymes [53], improving mitochondrial function [54,55], or altering the metabolic profile [56]. All these events might also be related to the anti-inflammatory mechanisms of flavonoids [57]. Our results showed that fisetin rescued mitochondrial respiratory capacity by upregulating the OXPHOS pathway and enhancing the expression of OXPHOS-related genes. On the other hand, fisetin ameliorated IFN-γ-enhanced glycolysis, as indicated by lactate concentration. These results showed that fisetin alleviated the aberrant metabolism in IFN-γ-stimulated macrophages, which may partly contribute to its anti-inflammatory effect.

Fisetin was found to selectively inhibit the MKK4-JNK1/2-AP-1 cascade to suppress the MAPK pathway in LPS-stimulated inflammation in our recent study [34]. The MAPK pathway has been reported to boost ISG transcription in macrophages [58], and impairment of the MAPK pathway inhibited the induction of ISGs [59]. On the other hand, IFN-γ is known to activate c-Jun-dependent AP-1 DNA binding in the iNOS promoter [60]. Therefore, we wondered whether fisetin suppressed IFN-γ-triggered inflammation partly through the inhibition of the MAPK pathway. The data from transcription factor inference showed that JUNB was obviously enriched in IFN-γ-induced DEGs; however, a time-course study on signaling demonstrated that the MKK4-JNK1/2-c-Jun cascade was activated only by LPS, not by IFN-γ. Therefore, we considered that the MAPK pathway might not be involved in IFN-γ-triggered inflammation.

The concentration of fisetin used in this study was determined based on previous research [21,22], indicating that the concentration currently used is clinically daily achievable. Administration of 21 mg/kg liposomal fisetin can maintain the plasma concentration of fisetin at around 6 μM over 4 h [61]. In a mouse experiment, this dose only delayed tumor growth at the early stage, while co-administered with cyclophosphamide significantly reduced tumor volume [61], which indicated the limitations of fisetin for direct clinical application. Therefore, we focused on the preventive effect and treated the cells with a low dose (5 μM) of fisetin to observe the DEGs to mimic the potential dose of daily consumption. We chose the threshold of a 1.3-fold increase or a 0.77-fold decrease for the DEGs with the criterion of *q* < 0.05 between the two groups. To avoid type A errors, we further performed GSEA, which used all the genes from RNA-Seq to clarify the defined pathway, rather than selected DEGs. Several relevant studies also used such a low threshold, like a 1.2-fold increase or a 0.83-fold decrease, to identify the DEGs [62,63]. Thus, we considered that our threshold criterion was suitable for discovering the effect and mechanism of fisetin on IFN-γ-stimulated mouse macrophages.

Mouse RAW264 cells were widely used to study immune function and inflammation due to their ability to perform essential macrophage functions and their rapid proliferation as well as ease of handling. Thus, in this study, we used this cell line to investigate the effects and molecular mechanisms of fisetin, and the data are reproducible using RAW264 cells from passages 4 to 15. The results revealed that fisetin could attenuate IFN-γ-stimulated macrophage inflammation and ameliorate the disrupted cellular metabolism. The Jak1/2-STAT1-IRF1 pathway was suggested to be involved in the molecular mechanisms. The data provided basic and initial results on the biological regulation function of fisetin. On the other hand, the findings need to be further clarified by IFN-γ-related *in vivo* models and human interventions, as cell culture research does not capture the full metabolic process after fisetin consumption and cannot fully represent the complexity of *in vivo* inflammatory responses. Additionally, the use of a single cell type (RAW264 macrophages) limits the generalizability of our findings to other immune cells or tissues.

## 5. Conclusions

Fisetin is a promising anti-inflammatory flavonoid that reduces IFN-γ-induced expression of pro-inflammatory genes and M1 marker genes and restores IFN-γ-disturbed metabolism. The Jak1/2-STAT1-IRF1 pathway was suggested to be involved in the molecular mechanisms. The results from cultured cells provide preliminary data on the biological regulation function of fisetin, but further studies in IFN-γ-related *in vivo* models or human interventions are necessary to validate these findings and assess their broader physiological relevance.

## Figures and Tables

**Table 1:** The statistical table of differentially expressed genes (IFN-γ/CON).

Fold Change	UP (IFN-γ/CON)	Fold Change	DOWN (IFN-γ/CON)
≥10,000	1	≤0.0001	-
10,000> to ≥1000	14	0.0001< to ≤0.001	-
1000> to ≥100	55	0.001< to ≤0.01	4
100> to ≥10	281	0.01< to ≤0.1	85
10> to ≥2	1122	0.1< to ≤0.5	1072
2> to ≥1.3	1721	0.5< to ≤0.769	1807
Total	3194	Total	2968

**Table 2:** The statistical table of differentially expressed genes (FisIFN-γ/IFN-γ).

Fold Change	UP (FisIFN-γ/IFN-γ)	Fold Change	DOWN (FisIFN-γ/IFN-γ)
≥100	1	≤0.01	-
100> to ≥10	5	0.01< to ≤0.1	2
10> to ≥2	63	0.1< to ≤0.5	54
2> to ≥1.3	412	0.5< to ≤0.769	407
Total	481	Total	463

## Data Availability

The data that support the findings of this study are available from the corresponding author.

## Supplementary Materials

The following supporting information can be downloaded at: https://www.mdpi.com/article/10.3390/antiox14020182/s1, Table S1: The information on antibodies used in this study; Figure S1: Western blots of total proteins and corresponding quantitative analysis from time-course experiments.

## Abbreviations

IFN-γ, interferon-gamma; Jak, Janus kinase; STAT, signal transducer and activator of transcription; IRF, IFN-regulatory factor; ISGs, interferon-stimulated genes; ISRE, interferon-stimulated response element; GAS, IFN-γ-activated site; RNA-Seq, RNA sequencing; DEGs, differentially expressed genes; FC, fold change; PCA, principal component analysis; KEGG, Kyoto encyclopedia of genes and genomes; GSEA, gene set enrichment analysis; LISA, epigenetic landscape in silico deletion analysis; OCR, oxygen consumption rate; OXPHOS, oxidative phosphorylation; iNOS, inducible nitric oxide synthase; COX-2, cyclooxygenase-2; IL, interleukin; NO, nitric oxide; LPS, lipopolysaccharide; MAPK, mitogen-activated protein kinase; MKK4, mitogen-activated protein kinase kinase 4; JNK, c-Jun NH2-terminal kinases; TBP, TATA-binding protein.
