# Supplementary material for: The Effects of Fisetin on Gene Expression Profile and Cellular Metabolism in IFN-γ-Stimulated Macrophage Inflammation
Source: Antioxidants (Basel). 2025 Feb 4;14(2):182. doi: 10.3390/antiox14020182 (PMC11852181; doi:10.3390/antiox14020182)
Supplement: Supplementary file 1 [file antioxidants-14-00182-s001.zip › antioxidants-3359631-supplementary.pdf]

Supplementary Table S1. The information on antibodies used in this study.

Antibodies	Source	Identifier	Dilute factor
iNOS	Cell Signaling Technology	13120	1:1000
COX-2	Santa Cruz Biotechnology	sc-1747	1:1000
p-SEK1/MKK4 (T261)	Cell Signaling Technology	9151	1:500
SEK1/MKK4	Cell Signaling Technology	9152	1:1000
p-SAPK/JNK (T183/Y185)	Cell Signaling Technology	9251	1:500
SAPK/JNK	Cell Signaling Technology	9252	1:1000
p-c-Jun (S73)	Cell Signaling Technology	9164	1:1000
c-Jun	Cell Signaling Technology	9162	1:1000
p-Jak1 (Y1034/1035)	Cell Signaling Technology	3331	1:1000
Jak1	Proteintech	66466-1-lg	1:2000
p-Jak2 (Y1007/1008)	Cell Signaling Technology	3771	1:500
Jak2	Santa Cruz Biotechnology	sc-390539	1:350
p-STAT1 (Y701)	Cell Signaling Technology	7649	1:1000
STAT1	Proteintech	66545-1-lg	1:10000
IRF1	Cell Signaling Technology	8478	1:1000
TBP	Proteintech	66166-1-lg	1:20000
α-Tubulin	Santa Cruz Biotechnology	sc-5286	1:2000

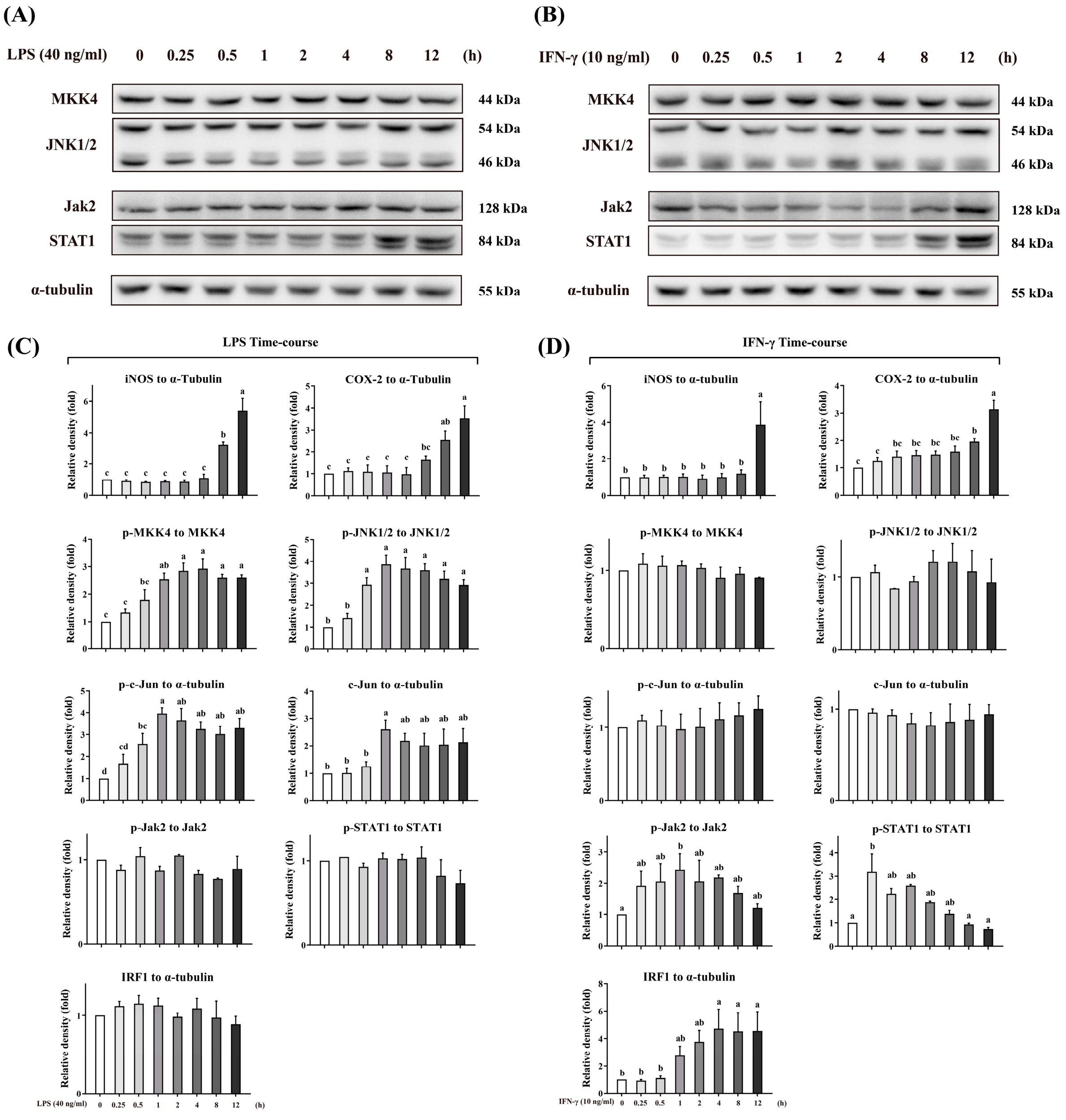

Supplementary Figure S1. Western blots of total proteins and corresponding quantitative analysis from time-course experiments. **(A,B)** Western blots of MKK4, JNK1/2, Jak2, and STAT1 in the time-course experiment by LPS stimulation **(A)** or IFN-γ stimulation **(B)**, corresponding to the results in Figure 7. **(C,D)** The relative quantitative graphs for the time-course experiment by LPS stimulation **(C)** or IFN-γ stimulation **(D)**. RAW264 cells were pre-cultured for 21 h and starved in serum-free medium for 2.5 h. The cells were then exposed to 40 ng/mL LPS or 10 ng/mL IFN-γ. Whole cell lysates were harvested after a defined stimulation time, and analyzed by Western blot assay. The relative density was calculated as the intensity of the treatment relative to that of the control normalized to α-tubulin or respective total proteins by densitometry. Each value represents the mean ± SD; different letters between groups indicate significant differences ($p < 0.05$). The blots presented are representatives from at least three independent experiments, using cells derived from at least two separate preparations.
